# Management of oligometastatic anal cancer—a literature review and a proposed treatment algorithm

**DOI:** 10.1016/j.esmogo.2025.100209

**Published:** 2025-07-22

**Authors:** A. Johnsson, K.L. Wind, M.P. Nilsson

**Affiliations:** 1 Department of Oncology, Skane University Hospital, Lund, Sweden; 2 Department of Experimental Clinical Oncology, Aarhus University Hospital, Aarhus, Denmark

**Keywords:** oligometastatic anal cancer, treatment algorithm, definitive metastasis-directed treatment

## Abstract

Oligometastatic squamous-cell carcinoma of the anus (omSCCA) is a very rare condition. The literature is sparse, and treatment guidelines are lacking. The aim of the present work was to carry out a literature review and to propose an algorithm for intensified treatment of patients with omSCCA. A PubMed search was carried out, which resulted in 15 relevant publications that included any type of definitive metastasis-directed therapy of mSCCA. All of the studies were retrospective. The local treatments were mostly surgical resections of liver metastases, but also radiofrequency ablation and radiotherapy were used, for metastases located in the liver, lungs, or other organs. The reported median overall survival across the studies ranged from 22 to 53 months. Three of the studies described extended-field chemoradiation of para-aortic lymph node metastases (M1 disease), suggesting long-term survival in up to half of the patients. Taken together, these results indicate that a subset of patients with omSCCA may benefit from a multimodal approach, including definitive metastasis-directed therapy given with curative intent. Based on the literature review and clinical experience, we propose a treatment algorithm, including metastasis-directed treatment as well as chemoradiation and induction chemotherapy. The choice of treatment and sequencing depends on the location and extent of the metastases and whether the metastases are synchronous or metachronous. In the absence of strong evidence, the proposed algorithm should be regarded as a starting point for discussions rather than detailed guidelines. Further studies and international collaborations are needed to optimize the treatment of omSCCA.

## Highlights

• omSCCA is very rare and treatment guidelines are lacking.
• A subset of omSCCA patients benefit from intensified therapy given with curative intent.
• Based on a literature review, a new treatment algorithm for omSCCA is proposed.
• Definitive metastasis-directed treatment should be considered in omSCCA.

## Introduction

Squamous-cell carcinoma of the anus (SCCA) is an uncommon malignancy. Most patients with SCCA have localized disease confined to the pelvic region, and standard treatment is chemoradiotherapy (CRT), which is usually effective, resulting in a complete remission rate of ∼90% and a 5-year overall survival (OS) of 70%-80%.^1^^,^^2^ Induction chemotherapy before CRT or maintenance chemotherapy delivered after CRT of localized SCCA has not proven beneficial.^3^, ^4^, ^5^ Distant metastases from anal cancer (mSCCA) occur in 15%-20% of the patients, either synchronously at the time of SCCA diagnosis or metachronously during follow-up, most commonly appearing in the liver, lungs, or extrapelvic lymph nodes. The randomized InterAACT trial has established carboplatin–paclitaxel as a standard first-line regimen in the palliative setting with an overall response rate of 59% and a median OS of 20 months.^6^ A phase II trial of a triplet chemotherapy regimen, mDCF [docetaxel, cisplatin, and 5-fluorouracil (5-FU)], has shown an overall response rate of 89%.^7^ Although initial response rates are high, most studies on palliative chemotherapy in mSCCA have shown a relatively poor long-term outcome with <30% 5-year OS.^1^^,^^8^

However, retrospective studies have shown that a subset of patients with limited distant metastases, known as oligometastatic SCCA (omSCCA), may benefit from a more aggressive approach, including surgical resection or other locally ablative treatments of the distant metastases. Some of these patients are even cured. There are no established guidelines on how to treat patients with omSCCA. As omSCCA consists of different clinical situations, treatment decisions are influenced by whether the metastases are synchronous or metachronous, as well as by the localization and extent of the metastases. The aim of the present work was to review the literature on omSCCA and to present an algorithm on how omSCCA could be treated with curative intent.

## Oligometastatic disease

Historically, metastatic cancer was considered an incurable disease. This notion was challenged by the results of retrospective studies, including mainly colorectal cancer^9^ and sarcoma patients with a limited number of liver or lung metastases,^10^ showing that a fair proportion of the patients (30%-40%) achieved long-term survival or even cure with radical surgery. Supported by the results of these early studies, the concept of oligometastatic disease (OMD) was first proposed in 1995.^11^ It represents an intermediate state between localized cancer and widespread metastatic disease. While surgery has since remained the gold standard for radical treatment of oligometastases, high-level evidence from randomized trials is lacking. However, other ablative interventions, in particular stereotactic body radiotherapy (SBRT), have been investigated in randomized trials over the past decade, summarized by Liu et al.^12^ The evidence for a survival benefit of SBRT in OMD is currently strongest for non-small-cell lung cancer, but other cancer diagnoses and histologies have been included in the phase II SBRT trials as well and phase III trials are ongoing.^12^ The SABR-COMET trial enrolled 99 patients with any solid tumor (no case of SCCA included) and one to five metastases located in the lung, bone, liver, adrenal gland, brain, or lymph nodes.^13^ Patients were randomized to standard-of-care systemic therapy ± SBRT to all metastases. With long-term follow-up, the 8-year progression-free survival was 21% versus 0% (*P* < 0.001) and 8-year OS was 27% versus 14% (*P* = 0.01), favoring the addition of metastasis-directed SBRT.

There is no uniform definition of OMD. In a European Society for Radiotherapy and Oncology (ESTRO)–American Society for Radiation Oncology (ASTRO) consensus document focusing on metastasis-directed radiotherapy, OMD was defined as not more than five metastases that could be safely treated with modern radiotherapy to potentially curative doses.^14^ The way of handling OMD varies considerably between different cancer types, depending on differences in tumor biology, sensitivity to radiotherapy, systemic treatments, etc. To the best of our knowledge, there has been no specific definition of omSCCA in the literature, but a pragmatic definition could be: ‘limited distant metastases of SCCA, where all metastatic deposits are suitable for radical local treatments such as surgical resection, other ablative techniques, or definitive radiotherapy’.

## Literature review

To identify available publications on a definitive treatment strategy for omSCCA, a systematic literature search in the PubMed database was carried out. On 30 May 2025, the final systematic search was conducted. The following filters were added: English language, studies including humans only, and publication date between 1 January 2000 and 30 May 2025. Only publications on squamous-cell histology were included, and case reports were excluded, whereas case series were accepted.

**Figure 1:**
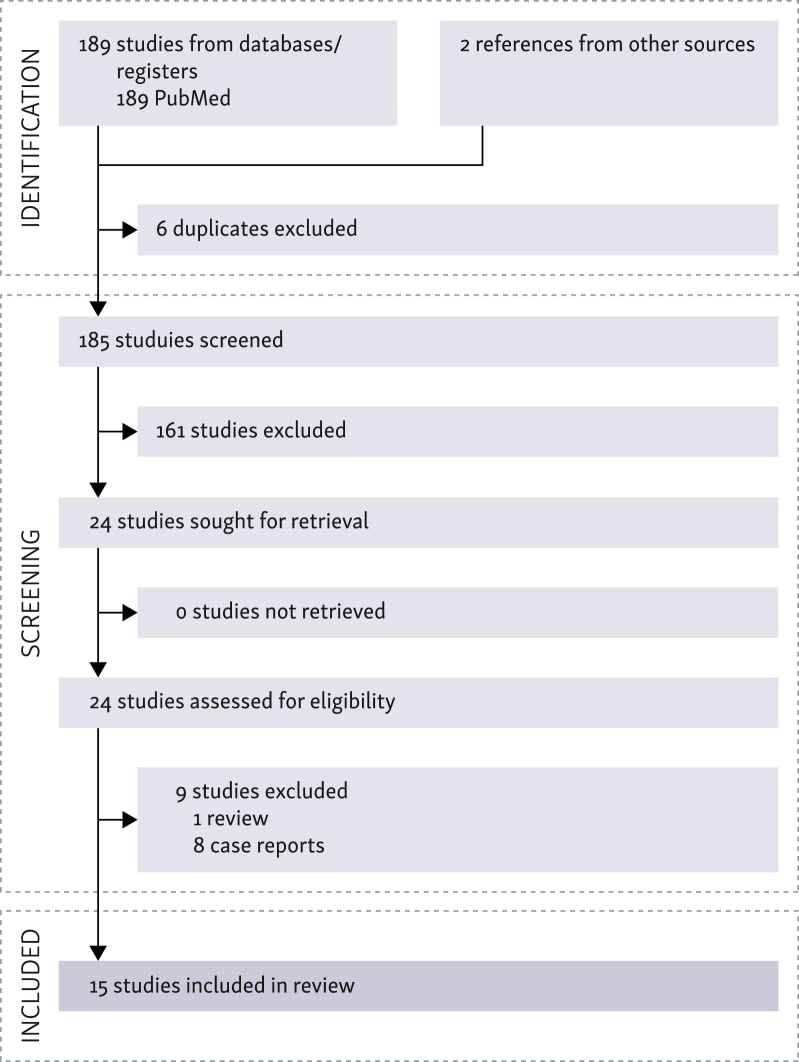
**PRISMA flow chart of literature review on oligometastatic anal cancer.** PRISMA, Preferred Reporting Items for Systematic Reviews and Meta-analyses.

All titles yielded from the search were screened, and relevant abstracts were selected for further reading. The first search string resulted in 165 titles. A second search string was conducted with a specific focus on para-aortic and common iliac lymph node metastases and resulted in nine titles. All abstracts were screened, but this search did not yield any new titles. Finally, a third search string focusing on liver resection and squamous-cell carcinoma was conducted and resulted in two additional publications. References of all relevant manuscripts were screened, and different combinations of the relevant search words were used to identify missing relevant publications, resulting in another two relevant publications. In total, 15 relevant publications were identified (Figure 1 and Table 1). Search strings are available as Supplementary Material, available at https://10.1016/j.esmogo.2025.100209.

**Table 1:** Literature on definitive treatment strategies in omSCCA

	Method, period, patients	Synchronous/metachronous	Locations of metastases	Metastases-directed therapy	Induction/adjuvant treatment	OS	Other
Organ metastases							
Rogers et al. 2023^15^	Retrospective 2018-2022 SCCA *n* = 8	Synchronous *n* = 4 Metachronous *n* = 4	Liver *n* = 8	Metastasectomy *n* = 8	Induction CT *n* = 8		1-year DFS = 56.2%
Lo et al. 2024^16^	Retrospective 2008-2022 SCCA *n* = 21	Synchronous *n* = 4 Metachronous *n* = 17	Liver *n* = 21	Metastasectomy *n* = 19 Ablation *n* = 1 Metastasectomy + ablation *n* = 1	Induction CT *n* = 10 Post-operative CT *n* = 9 Adjuvant RT *n* = 3	Median OS = 32.2 months 5-year OS = 50%	Median RFS = 7.7 months 5-year PFS = 30%
Grave et al. 2022^17^	Retrospective 2013-2018 SCCA *n* = 16 Stage IIIB *n* = 4 Stage IV *n* = 12	Synchronous *n* = 12	Liver *n* = 7 Lung *n* = 2 Bone *n* = 2 CI LN *n* = 1 Skin *n* = 1	Ablation *n* = 8 Metastasectomy *n* = 6 (liver) SBRT *n* = 1 Extended RT fields *n* = 2	Induction CT *n* = 16	3-year OS = 75%	Median PFS = 22.2 months 3-year PFS = 37.5% Median MFS = 34 months 3-year MFS = 48%
Wind et al. 2022^18^	Retrospective 2000-2018 SCCA *n* = 19	Synchronous *n* = 19	Liver *n* = 5 Lung *n* = 1 Bone *n* = 1 Skin *n* = 3 LN *n* = 9	Metastasectomy *n* = 3 Metastasectomy + RFA *n* = 1 RFA + SBRT *n* = 1 Extended RT fields *n* = 13	Induction CT *n* = 19	3-year OS = 74% 5-year OS = 55%	3-year DFS = 47% 5-year DFS = 41%
Engstrand et al. 2021^19^	Retrospective 2002-2019 SCC *n* = 45 SCCA *n* = 56	Synchronous *n* = 16 Metachronous *n* = 40	Liver *n* = 56	Metastasectomy *n* = 48 Ablation *n* = 2 Resection + ablation *n* = 6	Induction CT *n* = 27 Post-operative CT *n* = 7 Unspecified *n* = 1	SCCA: median OS = 50 months 5-year OS = 45%	SCCA: median RFS = 16 months
Goldner et al. 2020^20^	Retrospective 2004-2014 SCCA *n* = 165	NR	Liver *n* = 49 Lung *n* = 15 Bone *n* = 8	Metastasectomy *n* = 165	NR	Median OS = 23 months (liver-only median OS = 34 months, lung-only median OS = 15 months) 2-year OS = 48% 5-year OS = 28%	
Sclafani et al. 2019^21^	Retrospective 2008-2017 SCCA *n* = 8	Synchronous *n* = 2 Metachronous *n* = 6	Liver *n* = 5 Lung *n* = 3	Metastasectomy *n* = 7 RFA + RT *n* = 1	Induction CT *n* = 3	Median OS = 31 months	Median PFS = 4.5 months
Heinze et al. 2018^22^	Retrospective 2008-2016 SCCA *n* = 7	Synchronous *n* = 4 Metachronous *n* = 3	Liver *n* = 5 Lung *n* = 3 LN *n* = 1	IBA *n* = 7	Induction CT *n* = 6	Median OS = 25.2 months	Median PFS = 3.3 months LTC rate 97.4%
Evesque et al. 2017^23^	Retrospective 2000-2014 SCCA *n* = 50 Multimodality treatment *n* = 23 Standard CT *n* = 22	Synchronous *n* = 10 Metachronous *n* = 40	LN *n* = 16 Liver *n* = 22 Lung *n* = 4 Bone *n* = 6 Brain *n* = 1 Peritoneum *n* = 1	Metastasectomy *n* = 13 RT *n* = 11 RFA *n* = 6	None	Multimodality treatment: median OS = 22 months Standard CT treatment: median OS = 13 months	Multimodality treatment: median TFS = 10 months Standard CT treatment: median TFS = 5 months
Omichi et al. 2017^24^	Retrospective 1998-2015 SCC *n* = 28 SCCA *n* = 19	Synchronous *n* = 14 Metachronous *n* = 14	Liver *n* = 28	Metastasectomy *n* = 28	Induction CT *n* = 22 Post-operative CT *n* = 8	SCC: median OS = 33.3 months 3-year OS = 52% 5-year OS = 47%	SCC: median RFS = 9.3 months 3-year RFS = 25%
Eng et al. 2014^25^	Retrospective 2000-2012 SCCA *n* = 77 Multidisciplinary management *n* = 33 Palliative CT *n* = 44	NR	Liver *n* = 11 Lung *n* = 2 LN *n* = 5 Pelvis *n* = 5	Metastasectomy or RFA *n* = 19 CRT *n* = 14	Induction CT *n* = 33	Multidisciplinary: median OS = 53 months	Multidisciplinary: median PFS = 16 months
Pawlik et al. 2007^26^	Retrospective 1988-2006 SCC *n* = 52 SCCA *n* = 27	Synchronous *n* = 9 Metachronous *n* = 18	Liver *n* = 27	Metastasectomy *n* = 24 Metastasectomy + RFA *n* = 2 RFA *n* = 1	Induction CT *n* = 20 Post-operative CT *n* = 17	5-year OS = 22.9%	Median DFS = 9.6 months
Para-aortic lymph node metastases							
Nilsson et al. 2020^27^	Retrospective 2009-2017 SCCA *n* = 4	Synchronous *n* = 4	CI/PA LN *n* = 4	CRT with extended RT fields *n* = 4	None	NR	No recurrence with 22 months median follow-up
Holliday et al. 2018^28^	Retrospective 2002-2016 SCCA *n* = 30	Synchronous *n* = 30	PA LN *n* = 30	CRT with extended RT fields *n* = 30	None	3-year OS = 67%	3-year DFS = 42%
Hodges et al. 2009^29^	Retrospective 2001-2007 SCCA *n* = 6	Synchronous *n* = 6	PA LN *n* = 6	CRT with extended RT fields *n* = 6	None	3-year OS = 63%	3-year LR control = 100% 3-year distant control = 56%

CI, common iliac; CRT, chemoradiation; CT, chemotherapy; DFS, disease-free survival; IBA, interstitial brachytherapy; LN, lymph nodes; LR, local recurrence; LTC, local tumor control; MFS, metastasis-free survival; NR, not reported; omSCCA, oligometastatic squamous-cell carcinoma of the anus; OS, overall survival; PA, para-aortic; PFS, progression-free survival; RFA, radiofrequency ablation; RFS, recurrence-free survival; RT, radiotherapy; SCC, squamous-cell carcinoma; SCCA, squamous-cell carcinoma of the anus; TFS, time to failure of strategy.

### Organ metastases

Twelve publications included patients with organ metastases, mainly in the liver (Table 1). All of these studies were retrospective and generally small, with a median of 20 patients included (range 7-165 patients). The studies varied regarding information on, for example, the proportion of synchronous versus metachronous disease, induction/adjuvant chemotherapy regimens, sequencing of treatment modalities, and CRT dosing in case of synchronous mSCCA. The metastasis-directed local treatments were mostly surgical resections of liver metastases, but radiofrequency ablation and radiotherapy were also used. The reported median OS across the studies ranged from 22 to 53 months, and the 5-year OS rate ranged between 23% and 55%.

The study by Engstrand et al. included 102 patients with SCC and liver metastases, all treated with surgery.^19^ The 5-year OS was significantly better for SCCA (*n* = 56) than for SCC of other primary locations (45% versus 25%; *P* = 0.02).

Even though the data are limited, and cross-study comparisons should be avoided, a subpopulation of omSCCA seems to benefit from intensified treatment, including radical metastasis-directed treatment of distant metastases.

### Para-aortic lymph node metastases

Three studies reported outcomes for SCCA patients with para-aortic lymph node metastases treated with upfront extended-field CRT. The largest of these was a single-center retrospective analysis of 30 patients.^28^ The 3-year disease-free survival was 42%, and 3-year OS was 67%. Four patients had an out-of-field para-aortic recurrence as first failure, all of whom had a radiation field that extended less than one vertebral body above the most cranially located involved lymph node. The authors concluded that ‘extended-field CRT is a potentially curative treatment option for patients presenting with SCC of the anal canal with metastases limited to the para-aortic lymph nodes’. With modern radiotherapy techniques, the risk of severe toxicity during and after extended-field CRT is acceptable.^30^ The Nordic consensus guidelines for radiotherapy of para-aortic lymph node metastases in SCCA have recently been published.^31^

## Treatment algorithms

**Figure 2:**
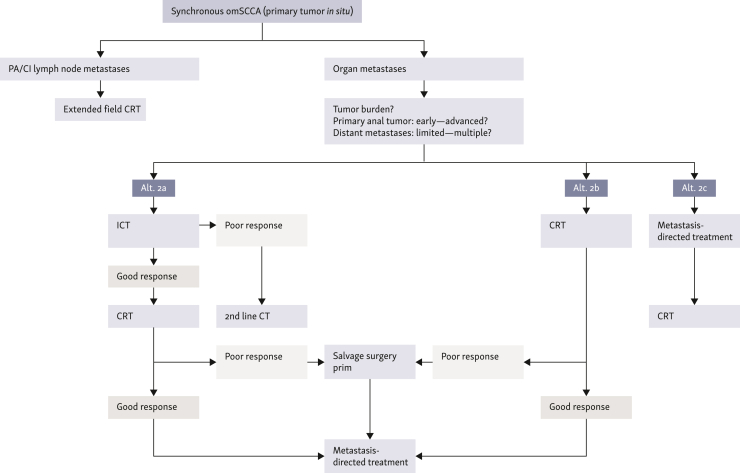
**Treatment algorithm for synchronous oligometastatic anal cancer (omSCCA).** Treatment alternatives 2a-2c, see text for explanation. Metastasis-directed therapy: surgical resection, stereotactic radiotherapy, or other ablative techniques. Alt., alternative; CRT, chemoradiotherapy of primary tumor + pelvic lymph nodes; CT, chemotherapy; ICT, induction chemotherapy; PA/CI, para-aortic/common iliac; salvage surgery prim, salvage surgery of primary anal tumor.

**Figure 3:**
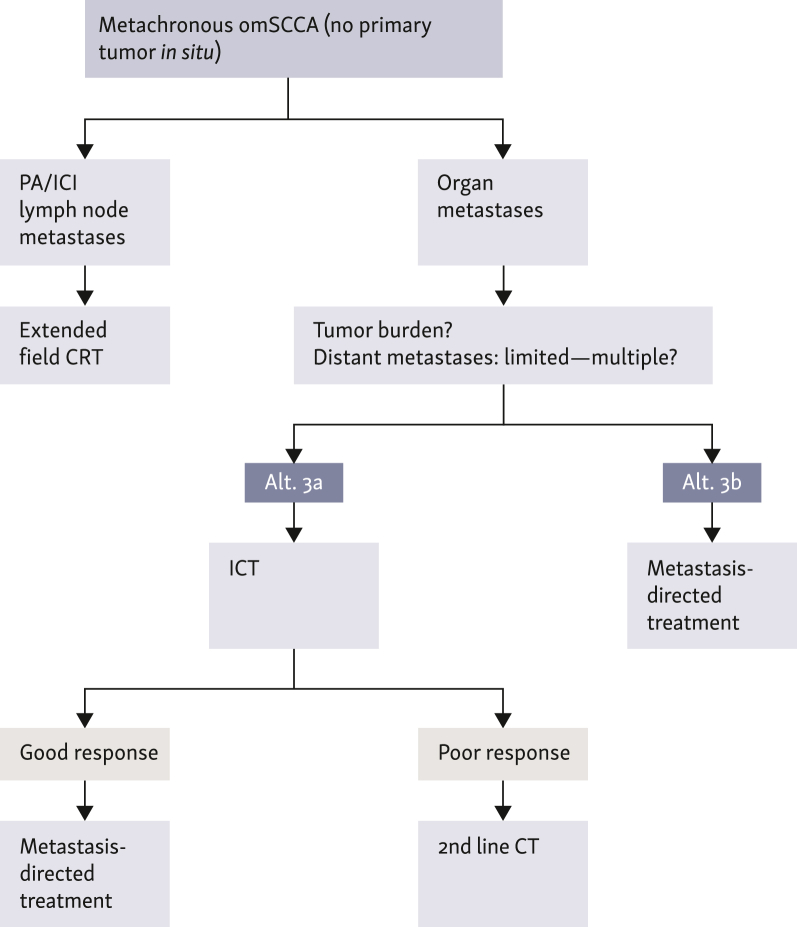
**Treatment algorithm for synchronous metachronous oligometastatic anal cancer (omSCCA).** Treatment alternatives 3a and 3b, see text for explanation. Metastasis-directed therapy: surgical resection, stereotactic radiotherapy, or other ablative techniques. Alt., alternative; CRT, chemoradiotherapy; CT, chemotherapy; ICT, induction chemotherapy; PA/CI, para-aortic/common iliac; salvage surgery prim, salvage surgery of primary anal tumor.

In Figures 2 and 3, suggested treatment algorithms are presented. The algorithms are divided into synchronous (primary tumor *in situ*) and metachronous (without primary tumor *in situ*) omSCCA, since they represent different clinical situations. Various treatment alternatives are given, where the tumor burden is taken into account, see below.

This is an intensive treatment that is delivered with curative intention. It requires that the patient is in good general condition, typically Eastern Cooperative Oncology Group performance status 0-1, and fit for the suggested interventions. The staging procedure should preferably include [^18^F]2-fluoro-2-deoxy-d-glucose–positron emission tomography with computed tomography (PET/CT), magnetic resonance imaging (MRI) of the liver (in case of liver metastases), and MRI of the pelvis (if synchronous disease). Multidisciplinary discussions are needed. Is the situation ‘truly oligometastatic’? Are all distant metastases accessible for local definitive treatments, upfront or after response to chemotherapy? What is currently most threatening for the patient? The primary tumor or the metastases?

### Treatment of synchronous omSCCA (organ metastases)

Treatment of synchronous omSCCA is a clinical challenge, where the patient besides the distant metastases also has a primary anal tumor that needs to be treated. The extent of both the primary tumor and the metastases may affect the choice of treatment sequencing. In case of multiple metastases, the treatment should start with chemotherapy, e.g. paclitaxel/carboplatin or mDCF (Figure 2, alternative 2a), where the latter could be preferred if the metastases are borderline resectable and a major response is needed. In case of good response to induction chemotherapy, CRT of the primary tumor should be carried out. The most widely used chemotherapy regimen in CRT is mitomycin combined with 5-FU or capecitabine, which could be used also in this situation. However, there are very few data on the efficacy of this regimen in mSCCA. Another option is to use cisplatin–5-FU, which is effective in mSCCA and also has radiosensitizing properties.

If CRT leads to local tumor control, metastasis-directed treatment (surgical resection, SBRT, or other ablative technique) should follow. Regarding the sequencing of CRT and local treatment of the metastases, individualized assessments are needed. If both the primary tumor and the distant metastases have responded well to induction chemotherapy, it may in some cases be better to give local treatment of the distant metastases before CRT of the primary tumor, since the latter is a prolonged and often toxic treatment, leading to a long delay before the local treatment of metastases can take place.

**Figure 4:**
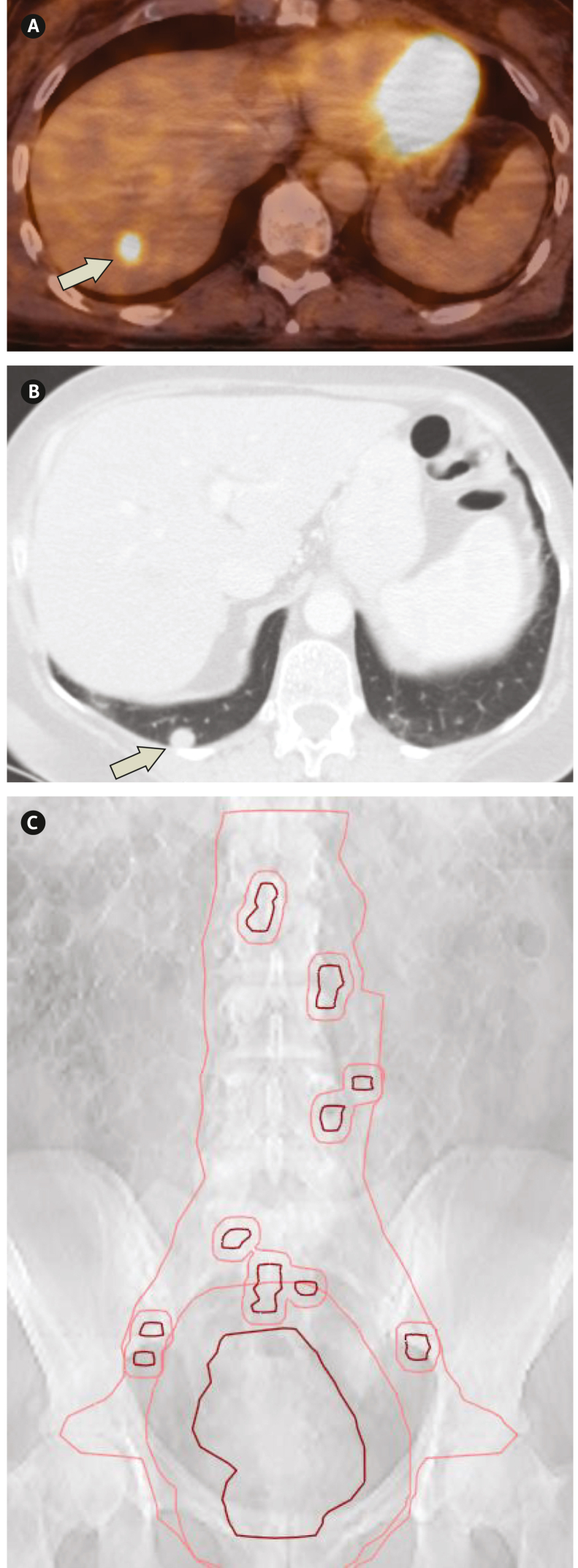
**Three patients with oligometastatic anal cancer treated with curative intent.** PET/CT of synchronous liver metastasis (A); CT of metachronous lung metastasis (B); topogram of radiotherapy planning gross tumor volume and clinical target volume for a patient with synchronous para-aortic lymph node metastases (C). CT, computed tomography; PET, positron emission tomography.

In case of very limited—e.g. a single metastasis in the liver or lung (Figure 4A and B)—metastatic spread, the treatment sequencing could be affected by the extent of the primary anal tumor. If the primary tumor is small (typically stage T1-2) and not causing major symptoms, local treatment of the metastasis could be done first if this is predicted to be an uncomplicated procedure (Figure 2, alternative 2c), followed by CRT of the primary tumor. If the primary tumor is advanced (T3-4) and there is a single metastasis, upfront CRT should be the preferred strategy (Figure 2, alternative 2b).

### Treatment of metachronous omSCCA (organ metastases)

For patients with metachronous omSCCA, the same evaluation of the metastatic burden as above is recommended. A single metastasis could be considered for local treatment upfront (Figure 3, alternative 3b), whereas patients with multiple metastases usually start with induction chemotherapy followed by local treatment of the metastases in case of good response (Figure 3, alternative 3a). Regarding adjuvant chemotherapy after radical local treatment of metastases, there are to the best of our knowledge no data supporting its value.

Patients with metachronous omSCCA with a residual anal tumor after CRT or a concurrent local recurrence are a real clinical challenge, where both salvage pelvic surgery and radical treatments of the metastases are required for the chance of cure. This situation is not included in the algorithm. Individualized management is needed. The prognosis is poor, and palliative treatment is sometimes the best option, which is beyond the scope of this paper.

### Treatment of para-aortic lymph node metastases

The recommended treatment of para-aortic lymph node metastases is upfront CRT, in the synchronous situation given as extended-field CRT (Figures 2, 3, and 4C).^31^ In the metachronous setting, CRT is given to the para-aortic lymph nodes only, with an elective clinical target volume covering lymph node stations down to the cranial border of the previous radiation fields. There is no evidence supporting the use of induction chemotherapy, but it may be considered in case of extensive and/or large para-aortic metastases that have a high risk of microscopic distant metastases or where tumor shrinkage by chemotherapy is deemed necessary to reduce the irradiated volume.

### Treatment of unusual omSCCA locations

Thus, the most common sites of distant metastases of SCCA are the liver, lung, and para-aortic lymph nodes, but occasionally omSCCA is located in other organs. Brain metastases from SCCA can be treated in the same way as brain metastases from other malignancies, i.e. surgery with post-operative stereotactic radiosurgery (SRS) or upfront SRS for a limited number of metastases and whole brain radiotherapy for multiple metastases. Isolated lymph node metastases in the mediastinum or the supraclavicular region could be treated with CRT or hypofractionated radiotherapy, including adjacent lymph node stations. Bone metastases could be treated with SBRT, but the chance of cure is most likely very small. An exception might be isolated sacral and pelvic bone metastases, as they could be caused by a locoregional spread of tumor cells via lymphatic vessels or the vertebral venous plexus rather than a distant spread, analogous to isolated sternal metastases in breast cancer.

## Discussion

omSCCA is a rare condition, and treatment guidelines are currently lacking. Based on a literature review and clinical experience, we propose an algorithm for the management of patients with omSCCA.

Results from retrospective studies have indicated that a portion of patients with OMD can be cured with metastasis-directed ablative therapies. Even though most of the current evidence is derived from surgical resection of liver and lung metastases in colorectal cancer and sarcomas, or SBRT in oligometastatic non-small-cell lung cancer, the concept of OMD has been used for an increasing number of cancer types over the past decade, including SCCA.

Firstly, we carried out a literature review on radical treatment of metastases in omSCCA. No prospective or randomized trials were identified. For organ metastases, we found 12 retrospective studies, mainly including patients with liver metastases treated with surgery. The 5-year OS in these studies was in the range of 30%-50%, which is similar to outcomes in other malignancies, e.g. colorectal cancer. Furthermore, in a multi-institutional study, 5-year OS following surgical resection of liver metastases was significantly better for SCCA patients than for patients with SCC of other primary sites.^19^ For para-aortic lymph node metastases, the results of three retrospective studies indicated that up to half of the patients could be cured with upfront extended-field CRT, which is equivalent to data from prospective studies in cervical cancer. Para-aortic lymph node metastases are currently classified as M1 in SCCA and are thus considered metastatic disease. The mechanism of tumor spread to the para-aortic region is however fundamentally different from the hematogenous spread of tumor cells to other organs. In SCCA, tumor cells usually spread via lymphatic routes in a predictable step-by-step fashion, and the common iliac and para-aortic lymph node regions are next in line following the iliac regions. To emphasize the regional rather than distant pattern of spread, it has been suggested that common iliac and para-aortic lymph node metastases in SCCA might more properly be staged as N2 and N3,^27^ which would be in line with the updated TNM9 (tumor–node–metastasis) classification of cervical cancer.^32^

Following the literature review, we propose a treatment algorithm for patients with omSCCA. The algorithm should be regarded as an attempt to structure the sequencing of different treatment modalities in different clinical situations. There is not enough evidence to provide complete and comprehensive guidelines, but our algorithm may constitute a valuable complement to the current National Comprehensive Cancer Network (NCCN) and European Society For Medical Oncology (ESMO) guidelines, where omSCCA is not specifically dealt with.

Even though the literature on omSCCA is limited, one recommendation can be made: patients with omSCCA who have a good performance status should be evaluated for intensified multidisciplinary treatment aiming at long-term survival and possible cure. All cases should be discussed at a multidisciplinary team meeting. The pre-treatment work-up is important. It should include a PET/CT, which is supported by a study, showing significantly better OS in patients who had undergone a PET/CT before extended-field CRT for patients with para-aortic omSCCA.^28^ A pelvic MRI should be carried out in synchronous disease and an MRI of the liver in case of liver metastases. The purpose is to determine whether the patient has a ‘true’ omSCCA and exclude patients from intensive treatment if they have a more widespread mSCCA.

In the proposed algorithm, several items could be discussed. The optimal sequencing of different interventions is unknown. A general feature of the algorithm is the stratification by tumor burden of the primary tumor and metastases, respectively. There is no evidence supporting this, but it seems reasonable from a general oncological point of view.

Most patients with omSCCA will receive chemotherapy at some point. In case of widespread distant metastases, chemotherapy is an uncontroversial treatment choice, but its role as induction treatment in omSCCA, where the distant metastases are accessible for local ablative treatments upfront and no shrinkage is needed, is unclear. In localized SCCA, two randomized studies have failed to show any benefit from induction cisplatin/5-FU before definitive CRT.^3^^,^^4^ The effect of induction chemotherapy in omSCCA remains to be studied. Regarding adjuvant chemotherapy in omSCCA, i.e. after radical ablation of metastases, no studies have been carried out. In localized SCCA, the randomized ACT-II trial did not show any benefit of maintenance chemotherapy with cisplatin/5-FU after CRT.^5^ Admittedly, that is a different clinical situation, but currently, there are no data supporting the use of adjuvant chemotherapy in the omSCCA setting.

The most widely used regimens for mSCCA today are carboplatin/paclitaxel and mDCF, which have not been compared head to head, but higher objective response rates have been reported for mDCF (89% versus 59%).^6^^,^^7^ Higher efficacy of triple than doublet chemotherapy is not surprising and in line with, for example, metastatic colorectal cancer, where 5-FU, leucovorin, oxaliplatin and irinotecan (FOLFOXIRI) is more effective than 5-FU, leucovorin and irinotecan (FOLFIRI).^33^ Best choice of chemotherapy regimen in omSCCA has not been determined, but if major shrinkage of the metastases is needed to facilitate radical treatment of the metastases, mDCF could be preferred. More recently, the first results from the randomized InterAACT2 trial were presented,^34^ showing an improved response rate and prolonged progression-free survival by adding the immune checkpoint inhibitor retifanlimab to carboplatin/paclitaxel. If this regimen reaches approval for treatment of mSCCA, it will become another valid option also in omSCCA in the future.

Regarding the choice of metastasis-directed treatment in omSCCA, most of the studies in our literature review focused on surgical metastasectomy of liver metastases. Today, there are also other ablative methods available, such as thermal ablation using radiofrequency or microwaves, irreversible electroporation ablation, or SBRT that can be utilized for the treatment of distant metastases. Randomized studies comparing different metastasis-directed treatments for OMD are lacking, but given the good local control reported with either technique, the decision on which to use will be based on anatomic location and local availability, rather than on firm evidence.

In summary, an algorithm for the treatment of omSCCA is proposed. A major problem is that omSCCA is extremely rare, and the literature on the subject is very sparse. We acknowledge that some of our recommendations do not rest on solid scientific ground. The algorithm should be regarded as a starting point for further discussions rather than definite guidelines.

Several questions regarding omSCCA remain unanswered, e.g. optimal sequencing of treatment modalities, best chemotherapy regimen, preferred ablative method, and selection of patients for intensified multidisciplinary treatment of omSCCA with curative intent. International collaboration is needed to carry out prospective studies, but pooling retrospective data from several institutions could also provide useful new knowledge on the management of patients with omSCCA.
