# Supplementary material for: Management of oligometastatic anal cancer—a literature review and a proposed treatment algorithm
Source: ESMO Gastrointest Oncol. 2025 Jul 22;13(Pt A):100209. doi: 10.1016/j.esmogo.2025.100209 (PMC13459591; doi:10.1016/j.esmogo.2025.100209)
Supplement: Supplementary file 1 [file mmc1.docx]

**Search strings:**

**Pubmed May 30, 2025:**

Filters added: English, Humans, from 2000/1/1 - 2025/5/30

**Search string 1:**

("Anus Neoplasms"[Mesh]) AND ("metasta*"[Title/Abstract] OR "oligometasta*"[Title/Abstract]) AND ("multidisciplinary"[Title/Abstract] OR "multimoda*"[Title/Abstract] OR "definitive"[Title/Abstract] OR "curative"[Title/Abstract] OR "metastasis directed"[Title/Abstract:~4] OR "metastases directed"[Title/Abstract:~4] OR "metastasectomy"[Title/Abstract] OR "liver resection"[Title/Abstract:~4])

**Search string 2:**

("Anus Neoplasms"[Mesh]) AND ("para-aortic"[Title/Abstract] OR "common iliac"[Title/Abstract] OR "retroperitoneal lymph nod*"[Title/Abstract]) AND ("Radiotherapy"[Mesh] OR "Chemoradiotherapy"[Mesh])

**Search string 3:**

("liver resection"[Title/Abstract]) AND ("squamous cell carcinoma" [Title/Abstract])

| **Search string 1: May 30, 2025** | | |
| --- | --- | --- |
| **Search** | **Query** | **Items found** |
| #15 | (("Anus Neoplasms"[Mesh]) AND (("metasta*"[Title/Abstract]) OR ("oligometasta*"[Title/Abstract])) AND (("multidisciplinary"[Title/Abstract]) OR ("multimoda*"[Title/Abstract]) OR ("definitive"[Title/Abstract]) OR ("curative"[Title/Abstract]) OR ("metastasis directed"[Title/Abstract:~4]) OR ("metastases directed"[Title/Abstract:~4]) OR ("metastasectomy"[Title/Abstract]) OR ("liver resection"[Title/Abstract:~4]))) AND (humans[Filter]) AND (english[Filter]) AND (2000:2025[pdat])  //  #5 AND #14 AND (humans[Filter]) AND (english[Filter]) AND (2000:2025[pdat]) | **165** |
|  |  |  |
| #14 | (("multidisciplinary"[Title/Abstract]) OR ("multimoda*"[Title/Abstract]) OR ("definitive"[Title/Abstract]) OR ("curative"[Title/Abstract]) OR ("metastasis directed"[Title/Abstract:~4])) OR ("metastases directed"[Title/Abstract:~4]) OR ("metastasectomy"[Title/Abstract]) OR ("liver resection"[Title/Abstract:~4]) AND (humans[Filter]) AND (english[Filter]) AND (2000:2025[pdat])  //  #6 OR #7 OR #8 OR #9 OR #10 OR #11 OR #12 OR #13 AND (humans[Filter]) AND (english[Filter]) AND (2000:2025[pdat]) | **275118** |
| #13 | ("liver resection"[Title/Abstract:~4]) AND (humans[Filter]) AND (english[Filter]) AND (2000:2025[pdat]) | **12300** |
| #12 | ("metastasectomy"[Title/Abstract]) AND (humans[Filter]) AND (english[Filter]) AND (2000:2025[pdat]) | **2155** |
| #11 | ("metastases directed"[Title/Abstract:~4]) AND (humans[Filter]) AND (english[Filter]) AND (2000:2025[pdat]) | **148** |
| #10 | ("metastasis directed"[Title/Abstract:~4]) AND (humans[Filter]) AND (english[Filter]) AND (2000:2025[pdat]) | **467** |
| #9 | ("curative"[Title/Abstract]) AND (humans[Filter]) AND (english[Filter]) AND (2000:2025[pdat]) | **54329** |
| #8 | ("definitive"[Title/Abstract]) AND (humans[Filter]) AND (english[Filter]) AND (2000:2025[pdat]) | **68131** |
| #7 | ("multimoda*"[Title/Abstract]) AND (humans[Filter]) AND (english[Filter]) AND (2000:2025[pdat]) | **55029** |
| #6 | ("multidisciplinary"[Title/Abstract]) AND (humans[Filter]) AND (english[Filter]) AND (2000:2025[pdat]) | **94285** |
|  |  |  |
| #5 | ("Anus Neoplasms"[Mesh]) AND (("metasta*”[Title/Abstract]) OR ("oligometasta*"[Title/Abstract])) AND (humans[Filter]) AND (english[Filter]) AND (2000:2025[pdat])  /  (#1 AND #4 AND (humans[Filter]) AND (english[Filter]) AND (2000:2025[pdat])) | **631** |
|  |  |  |
| #4 | (("metasta*”[Title/Abstract]) OR ("oligometasta*"[Title/Abstract])) AND (humans[Filter]) AND (english[Filter]) AND (2000:2025[pdat])  //  #2 OR #3 ])) AND (humans[Filter]) AND (english[Filter]) AND (2000:2025[pdat]) | **390283** |
| #3 | ("oligometasta*"[Title/Abstract]) AND (humans[Filter]) AND (english[Filter]) AND (2000:2025[pdat]) | **2895** |
| #2 | (“metasta*"[Title/Abstract]) AND (humans[Filter]) AND (english[Filter]) AND (2000:2025[pdat]) | **389634** |
|  |  |  |
| #1 | ("Anus Neoplasms"[Mesh]) AND (humans[Filter]) AND (english[Filter]) AND (2000:2025[pdat]) | **4301** |

| **Search string 2: May 30, 2025** | | |
| --- | --- | --- |
| **Search** | **Query** | **Items found** |
| #11 | ("Anus Neoplasms"[Mesh]) AND (("Lymphatic Metastasis"[Mesh]) OR ("para-aortic"[Title/Abstract]) OR ("common iliac"[Title/Abstract) OR ("retroperitoneal lymph nod*"[Title/Abstract])) AND (("Radiotherapy"[Mesh]) OR ("Chemoradiotherapy"[Mesh])) AND ((humans[Filter]) AND (english[Filter])) AND (2000:2025[pdat])  //  #7 AND #10 AND ((humans[Filter]) AND (english[Filter])) AND (2000:2025[pdat]) | **66** |
|  |  |  |
| #10 | ("Radiotherapy"[Mesh]) OR ("Chemoradiotherapy"[Mesh]) AND (humans[Filter]) AND (english[Filter]) AND (2000:2025[pdat])  //  #8 OR #9 AND (humans[Filter]) AND (english[Filter]) AND (2000:2025[pdat]) | **127418** |
| #9 | ("Chemoradiotherapy"[Mesh]) AND (humans[Filter]) AND (english[Filter]) AND (2000:2025[pdat]) | **20547** |
| #8 | ("Radiotherapy"[Mesh]) AND (humans[Filter]) AND (english[Filter]) AND (2000:2025[pdat]) | **127418** |
|  |  |  |
| #7 | (("Anus Neoplasms"[Mesh]) AND (("Lymphatic Metastasis"[Mesh]) OR ("para-aortic"[Title/Abstract]) OR ("common iliac"[Title/Abstract) OR ("retroperitoneal lymph nod*"[Title/Abstract]))) AND (humans[Filter]) AND (english[Filter]) AND (2000:2025[pdat])  //  #1 AND #6 AND (humans[Filter]) AND (english[Filter]) AND (2000:2025[pdat]) | **188** |
|  |  |  |
| #6 | (("Lymphatic Metastasis"[Mesh]) OR ("para-aortic"[Title/Abstract]) OR ("common iliac"[Title/Abstract) OR ("retroperitoneal lymph nod*"[Title/Abstract])) AND (humans[Filter]) AND (english[Filter]) AND (2000:2025[pdat])  //  #2 OR #3 OR #4 OR #5 AND (humans[Filter]) AND (english[Filter]) AND (2000:2025[pdat]) | **64544** |
| #5 | ("retroperitoneal lymph nod*"[Title/Abstract]) AND (humans[Filter]) AND (english[Filter]) AND (2000:2025[pdat]) | **1562** |
| #4 | ("common iliac" [Title/Abstract]) AND (humans[Filter]) AND (english[Filter]) AND (2000:2025[pdat]) | **2921** |
| #3 | ("para-aortic"[Title/Abstract]) AND (humans[Filter]) AND (english[Filter]) AND (2000:2025[pdat]) | **2778** |
| #2 | ("Lymphatic Metastasis"[Mesh]) AND (humans[Filter]) AND (english[Filter]) AND (2000:2025[pdat]) | **59191** |
|  |  |  |
| #1 | ("Anus Neoplasms"[Mesh]) AND (humans[Filter]) AND (english[Filter]) AND (2000:2025[pdat]) | **4301** |

| **Search string 3: May 30, 2025** | | |
| --- | --- | --- |
| **Search** | **Query** | **Items found** |
| #4 | ("liver resection"[Title/Abstract]) AND ("squamous cell carcinoma" [Title/Abstract]) AND (humans[Filter]) AND (english[Filter]) AND (2000:2025[pdat])  //  #1 AND #2 AND (humans[Filter]) AND (english[Filter]) AND (2000:2025[pdat]) | **15** |
|  |  |  |
| #3 | ("squamous cell carcinoma" [Title/Abstract]) AND (humans[Filter]) AND (english[Filter]) AND (2000:2025[pdat]) | **76857** |
|  |  |  |
| #1 | ("liver resection"[Title/Abstract]) AND (humans[Filter]) AND (english[Filter]) AND (2000:2025[pdat]) | **8684** |

|  | **Search string 1** | **Search string 2** | **Search string 3** | **Other sources** |
| --- | --- | --- | --- | --- |
|  | N = 165 (May 30, 2025) | N = 9 (May 30, 2025) | N = 15 (May 27 2025) | N = 2 |
| **Lo 2024** | √ |  | √ |  |
| **Rogers 2023** |  |  |  | √ |
| **Grave 2022** |  |  |  | √ |
| **Wind 2021** | √ |  |  |  |
| **Engstad 2021** |  |  | √ |  |
| **Goldner 2020** | √ |  |  |  |
| **Sclafani 2019** | √ |  |  |  |
| **Heinze 2018** | √ |  |  |  |
| **Evesques 2017** | √ |  |  |  |
| **Omichi 2017** |  |  | √ |  |
| **Eng 2014** | √ |  |  |  |
| **Pawlik 2007** | √ |  |  |  |
| **Nilsson 2020** | √ | √ |  |  |
| **Holliday 2018** | √ | √ |  |  |
| **Hodges 2009** | √ | √ |  |  |
